# Comparative Study on the Microleakage of Three Root Canal Sealers

**Published:** 2011-02-15

**Authors:** Hengameh Akhavan, Farhad Zahdabadi, Peyman Mehrvarzfar, Anahid Ahmadi Birjandi

**Affiliations:** 1 *Department of Endodontics, Dental School, Islamic Azad University of Medical Science, Tehran, Iran*; 2 *Private Practice, Tehran, Iran*

**Keywords:** Apical Microleakage, Canal Sealer, Fluid Filtration, Root Canal Obturation

## Abstract

**INTRODUCTION:** The aim of this study was to examine and compare the apical sealing ability of AH26, AH Plus and AH Plus Jet using the fluid filtration model.

**MATERIALS AND METHODS:** In this experimental study, 70 single-rooted teeth were dissected from the cement-enamel junction. Canals were prepared with ProTaper rotary system and hand K-files and irrigated with 5.25% NaOCl and 17% EDTA. Ten teeth were assigned to the control group and divided into 5 positive and 5 negative controls. Remaining specimens were divided into 3 groups of 20 samples each and filled with gutta percha by lateral condensation technique. Each sample group was filled with either AH26, AH Plus or AH Plus Jet. Microleakage was assessed on the 2^nd^ and 30^th^ day by the amount of air bubble movement within the capillary glass tube connected to the root. One-way ANOVA test was used for analysis.

**RESULTS:** AH Plus Jet had the least microleakage value and AH Plus presented the highest rate; however, the differences were not statistically significant.

**CONCLUSION:** Under the conditions of this study, all three studied sealers provided satisfactory seal within the two time intervals. AH Plus Jet demonstrated slightly lower microleakage values; therefore, its application can be recommended in endodontic therapy.

## INTRODUCTION

**M**icroleakage of endodontically treated teeth is a major cause of treatment failure. A good apical seal plays critical role in the success of endodontic treatment. Accurate seal of the root canal is a difficult and sensitive task due to attributed root anatomy variations and accessory canals. Ingle *et al.* reported that 60% of the endodontic failures are due to incomplete and inappropriate obturations (1). Other studies have revealed that inadequate flow of gutta percha and its inability to adhere to dentinal walls leads to an insufficient seal (2,3). Subsequent to the introduction of new sealers into the market, various materials and methods have been investigated to improve and compare the sealing abilities of root canal materials. The results have been inconclusive; no sealer produced to date can accomplish all the requirements for a perfect root canal seal (4-6).

Cobankara *et al.* studied apical sealing ability of Rocanal 2, Sealapex, AH Plus and RC sealer via computerized fluid filtration; Sealapex provided a better seal compared to other sealers (7). Another study evaluated the fluid transport along gutta percha in canals filled with/without sealer AH26, Roekoseal Automix (RSA) and Pulp Canal Sealer (EWT) (5). Results showed that samples without sealer had the highest rate of fluid transport (leakage) compared to other groups. Another study reported greater micro-leakage for AH Plus compared to AH26 (4).

Pécora *et al.* studied the adhesion of root canal sealers to dentine via Er:YAG and AH Plus appeared superior to AH26 (8). The sealing ability of AH Plus, AH26 and RSA using dye penetration in teeth filled with lateral condensation or Thermafill method was also evaluated. Results showed that teeth obturated with Thermafill technique without sealer had the highest rate of dye penetration. However no statistical difference was observed between the mean apical dye penetrations among the three different sealers (9).

No significant difference in the sealing ability of RSA, Topseal and Endometason assessed by either clearing or cross section method was reported (10). Epoxy-resin based sealers are famous for their adhesive ability (10-12). AH26 is an epoxy-resin based material with good sealing ability even when it is solely used as the root canal filling (13). The long setting time and flowability of this material inhibits crack formation and fast detachment from the dentinal wall (14). It can harden in the presence of moisture, has high tissue compatibility and less than 0.5% constriction when entering the accessory canals (14,15). However, the release of formaldehyde and its long setting time (4 weeks) are unfortunate disadvantages (16). AH Plus is claimed to have the advantages of AH 26 but without releasing formaldehyde and with a shorter setting time. Moreover, it appears to be more radiopaque and have less microleakage compared to AH26 (17,18). AH Plus Jet is a new form of AH Plus, available in mixing syringes which can be directly injected into canal orifices. The adjustable syringe tip makes its use effective and infection control friendly (19).

The aim of this study was to evaluate and compare the sealing ability of AH26, AH Plus and AH Plus Jet via fluid filtration method after 2 and 30 days.

## MATERIALS AND METHODS

This *in vitro* experiment consisted of 70 extracted single-rooted, single-canalled incisors. The inclusion criteria included single straight canal with the apical curvature ≤20 degree (Schneider method), apical foramen ≤K-file #20, mature and sound apex (microscopically), and root canal patency. Exclusion criteria were: root decay, calcified canals (radiographically), root crack (radiographically and microscopically), and internal/external root resorption (radiographically).

Samples were washed and cleansed with a tooth brush under running water and preserved in 0.5% sodium hypochlorite solution. Tooth crowns were cut at CEJ with diamond disks (D&Z, Germany) to facilitate canal preparation; samples were then placed in 0.9% normal saline prior to the study. All the samples were prepared with ProTaper rotary system (Dentsply, Switzerland). Apical foramen was evaluated with master apical file (MAF). The canals were irrigated with 17% EDTA for 5 minutes and then 5.25% sodium hypochlorite followed by saline and distilled water. Five teeth were assigned to the positive control group; using lateral condensation technique, obturated with gutta percha without using sealer. The negative control group consisted of 5 teeth with liquid glue-covered apices and the tooth surfaces coated with 2 layers of nail varnish. The 60 remaining teeth were randomly divided into three experimental groups of 20 samples contained either AH26 (DENTSPLY, Switzerland), AH Plus (DENTSPLY, Switzerland) or AH Plus Jet (DENTSPLY, Switzerland) as the sealer. The quality of root canal treatments were assessed by parallel radiography. All the samples were then preserved in a 37^°^C and 100% humidity incubator for 48 hours.

**Figure 1:**
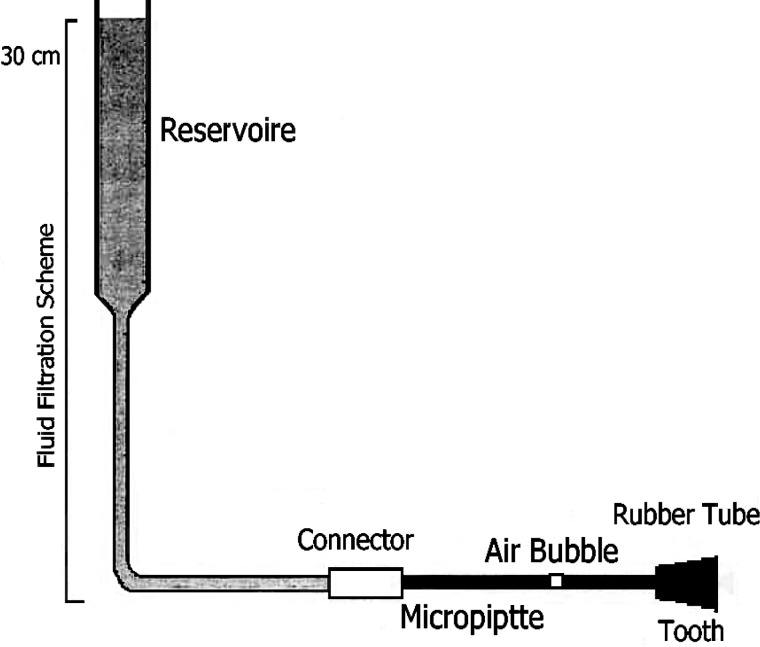
View of the fluid filtration model

To evaluate microleakage, samples were placed in a fluid filtration model. The model consisted of pre-measured 0.02mL micropipette (Germany) connected to a columnar reservoir with 3cm diameter via a polyethylene connector (Figure 1). The reservoir, connectors and the remaining pipes were filled with distilled water. Water altitude in the reservoir was 30 cm higher than the position of teeth apices in the rubber tube and hence provided a positive pressure of 16mm-Hg to guide the liquid towards root apices. Samples were separately placed in a rubber tube. The space between the root and the rubber tube was sealed with glue wax. An air bubble was incorporated into the pipette with an insulin syringe. The rubber tube was then filled with water and connected to the pipette and tube. The seal of all connecting areas were double checked. The model was horizontally oriented for 8 minutes for each sample. The movement of the air bubble was later measured and evaluated by the operator using a precise ruler and magnifier. Volume of air bubble movement was assessed according to the diameter of tube.

**Table 1:** The mean (±SD) microleakage in study groups at the two intervals

**Interval** **Sealer**	**N**	**2** ^nd^ ** Day**	**30** ^th^ ** Day**
**AH26 **	20	1.35±0.19	1.23±0.16
**AH Plus **	20	1.50±0.27	1.39±0.27
**AH Plus Jet **	20	0.97±0.09	0.85±0.08
**P value**		0.26	0.10

Results were then recorded in line with a micro liter (µL) scale. Data were analyzed with one-way ANOVA test. The significant level was set at α=0.05.

## RESULTS

The negative control group displayed no movement of air bubble in the model, indicating zero leakage. In the positive control group, the considerable displacement of air bubble revealed 10µL of microleakage within 8 minutes. This result approved the accuracy of study model.

One-way ANOVA test was used to compare the microleakage on the 2^nd^ and 30^th^ days; they revealed no significant difference between three study groups. The average microleakage difference between the 2^nd^ and 30^th ^day was assessed by Kruskal-Wallis test and revealed no significant difference.

The mean of microleakage on two time intervals within each group was assessed by paired t-test. Results are shown in Table1.

## DISCUSSION

The current study revealed that AH Plus Jet had the least leakage on the 2^nd^ and 30^th^ day; whereas AH Plus revealed the highest microleakage rate. There was no statistically significant difference between the leakage of the studied sealers.

Currently the most popular method used for microleakage assessment is fluid filtration which was first introduced by Wu *et al., *having many advantages over the dye penetration method (14).

Alteration of tooth structure after sectioning in dye penetration method makes sample re- evaluation impossible. Probability of bias due to multiple stages of tooth preparation and difficult evaluation of dye penetration between gutta percha and canal walls in the sectioned parts are other disadvantages (13). In the fluid filtration assessment method, modification of tooth structure is minimized and long term re-evaluation of samples is possible. The technique is simple, less time-consuming and provides possibility of microleakage assessment of individual samples in different observation periods. Detailed evaluation of microleakage by micro liter scale is also possible. Some obstacles exist in this method such as sealing of the space between root and the plastic tube which is important for bias prevention; therefore glue wax was used to seal this area in the current study. Precise measurement of the air bubble movement in the micropipette was carried out by an accurate ruler and a magnifier. Two operators double checked the results. The surface porosity of endodontic sealers is affected by mixing method. This applies to AH Plus Jet which is user friendly during mixing.

Zemner *et al.* compared the sealing ability of AH Plus and AH26 in teeth which were obturated using lateral condensation technique (11). The Microleakage was assessed using dye penetration after 2, 4 and 10 days. AH Plus demonstrated significantly more leakage compared to AH26. The fast setting of AH Plus and subsequent setting shrinkage might be the reason for this difference. AH26 has also been shown to have larger initial expansion compared to AH Plus (20). A further study compared microleakage of AH26, AH Plus, Diaket, Apexit and Ketac-endo by fluid filtration on 60 obturated teeth. AH Plus had greater leakage compared to AH26 within the first 24 hours after obturation; the difference was statistically insignificant (4). De Moore and De Bruyne assessed the long-term sealing ability of AH Plus and AH26 in 940 teeth obturated with lateral condensation, hybrid or thermafill techniques. Coronal and apical leakage was assessed separately via dye penetration consecutively after 1 day, 1 week, 2 weeks, one month and six months. Apical leakage of AH Plus was consistently higher compared to AH26; however, the difference was statistically insignificant. They reported that AH26 and AH Plus resulted in comparable sealing ability at all evaluation times when used with identical obturation techniques (21). The leakage study of Hollanda *et al.* on a split chamber design with BMI infusion displayed no significant difference between AH Plus and AH 26; however the agar diffusion test demonstrated AH Plus to have larger microbial inhibition zones compared with AH26 (22). Interestingly another study showed an absence of difference in the bacterial penetration in AH26, AH Plus, Seal apex and Ketac-Endo at 30 and 60 days. The reason might be due the study method and assessment technique (23). A further fluid filtration study revealed AH Plus and experimental MBP (a resin based sealer containing calcium hydroxide) to have lower leakage after 60 days compared to EndoREZ (24).The inconsistency with the result of our study might be due to the variance in study design and obturation techniques.

Tunga and Bodrumlu studied the sealing ability of epiphany resilion and AH Plus, using fluid filtration method 48 hours after obturation (n=54 teeth) (25). Their study only analyzed short term seal, unlike our study, and found no significant difference.

## CONCLUSION

AH Plus Jet had the least leakage during the course of the study, however all sealers can be regarded suitable for clinical use. Considering the novelty of AH Plus Jet, further research in this field is required to prove its efficacy.
